# Assessment of Physicochemical Parameters and Contaminants in Herbal Dietary Supplements Used in the Treatment of Inflammatory Bowel Disease

**DOI:** 10.3390/ph16060893

**Published:** 2023-06-18

**Authors:** Daniela Amidžić Klarić, Jelena Kovačić, Mario-Livio Jeličić, Snježana Zubčić, Vladimir Stankov, Marija Gulan Čičak, Boris Bučar, Ilija Klarić, Ana Mornar

**Affiliations:** 1 Faculty of Pharmacy and Biochemistry, University of Zagreb, A. Kovačića 1, 10 000 Zagreb, Croatia; damidzic@pharma.hr (D.A.K.); jkovacic@pharma.hr (J.K.); mljelicic@pharma.hr (M.-L.J.); mgulan@pharma.hr (M.G.Č.); 2 Agency for Medicinal Products and Medicinal Devices of Croatia, Ksaverska Cesta 4, 10 000 Zagreb, Croatia; snjezana.zubcic@halmed.hr; 3 Sample Control, Puškarićeva Ulica 18, 10 250 Lučko, Croatia; vladimir.stankov@sample-control.hr (V.S.); boris.bucar@sample-control.hr (B.B.); 4 Public Health Brčko DC, R. Dž. Čauševića 1, 76000 Brčko DC, Bosnia and Hercegovina

**Keywords:** dietary supplements, inflammatory bowel disease, USP, ethylene oxide, gluten, turmeric, Indian frankincense, green chiretta, black pepper

## Abstract

Inflammatory bowel disease is a complex disorder characterized by chronic gastrointestinal inflammation. Thus, patients prefer to use herbal dietary supplements containing turmeric, Indian frankincense, green chiretta, and black pepper in an attempt to cope better with their chronic condition. The dietary supplements’ dosage forms and herbal ingredients were assessed in terms of the products’ physicochemical parameters (weight uniformity, friability, disintegration, rupture test, tablet’s breaking force, and powder flowability) in view of the USP-NF requirements. In addition, contaminants such as organic solvents and ethylene oxide were evaluated using gas chromatography. Assessment of gluten via an Enzyme-Linked Immunosorbent Assay was also performed. Most of the products met USP requirements. The high average weight of one multicomponent tablet sample with a high breaking force value can explain the observed negative results of the disintegration test. A total of 26% of samples tested positive for gluten, but the most alarming fact is that the ethylene oxide levels found in two samples were up to 30 times higher than the EU limit. Accordingly, dietary supplement quality control is of fundamental importance.

## 1. Introduction

Dietary supplements comprise a broad array of products containing vitamins, minerals, amino acids, enzymes, and herbs available in forms such as tablets, capsules, and liquids added to the diet to benefit health. The global dietary supplement marketplace was valued at more than 191 billion USD in 2020 and is expected to reach more than 307 billion USD by 2028 [1]. This increase in the use of dietary supplements is particularly common in patients with chronic diseases, such as inflammatory bowel disease (IBD). This disease is a complex, life-threatening, refractory disorder characterized by chronic gastrointestinal inflammation. The term includes three different clinical states: Crohn’s disease (CD), ulcerative colitis (UC), and other conditions. IBD is characterized by states of exacerbation and remission, and the primary therapeutic goal is to maintain the possibly long remission phase [2,3]. 

Patients with IBD prefer to use herbal dietary supplements to cope better with their chronic condition, which includes episodes of abdominal pain, diarrhea, bloody stools, and weight loss. Turmeric (*Curcuma longa* L., *Zingiberaceae*) is a spice known for its bright yellow hue and citrus flavor; however, its polyphenols, curcuminoids, have gained significant attention among healthcare professionals and IBD patients for their anti-inflammatory effects. Indian frankincense (*Boswellia serrata* Roxb. ex Colebr., *Burseraceae*) belongs to the family of trees producing resin composed of groups of pentacyclic triterpene acids known as boswellic acids and has been shown to exert therapeutic effects in the IBD setting. Green chiretta (*Andrographis paniculata (Burm. f.)* Wall. ex Nees, *Acanthacea*), known as the ˝king of bitters˝, is widely used as *Kiriyattu* in Ayurveda medicine in India, and its main components, andrographolides, have been scrutinized with the modern drug discovery approach for anti-inflammatory benefits in a variety of inflammatory disease models, such as IBD. Finally, among phytochemicals, it is worth mentioning black pepper (*Piper nigrum* L., *Piperaceae*) extract, which has been under the spotlight for its therapeutic potential in IBD as well as its main ingredient, piperine, as a booster of the absorption of curcuminoids [4,5,6,7].

The quality issues associated with dietary supplements have received steady warnings from scientific and healthcare communities [8]. Risks include the absence or non-traceability of biologically active ingredients; synthetic substance adulteration; and the presence of harmful agents such as organic solvents, pesticides, fungicides, microbiological agents, metal impurities, and prescription-only pharmaceuticals [9]. To ensure the quality and safety of dietary supplements, the U.S. Food and Drug Administration (FDA) issued the Current Good Manufacturing Practices in Manufacturing, Packaging, Labelling, or Holding Operations for Dietary Supplements. In addition, the United States Pharmacopeia and the National Formulary (USP-NF) provide comprehensive laboratory testing for conformance to dietary supplement quality [10]. Still, an increasing number of health emergencies from unsafe dietary supplements, warning letters issued by the regulatory authorities for manufacturing violations, numerous publications documented variability in product ingredients versus label claims, and the lack of uniformity of product and raw material quality point to the fact that routine and stringent quality assurance testing has been lacking and, in some instances, not performed [11,12,13,14,15,16]. With this in mind, we believe that sustainable quality control of dietary supplements can only be based on up-to-date multiple analytical methods to ensure the high quality and safety of these products and their raw materials.

In our previous study, we demonstrated and applied the first developed method for the simultaneous analysis of various active ingredients present in the dietary supplement and raw material products based on turmeric, Indian frankincense, green chiretta, and black pepper, which are on the rise today for the treatment of IBD [17]. 

In the present study, the above-mentioned dietary supplements manufactured in solid and liquid dosage forms, as well as dietary supplement ingredients as raw material, were assessed in terms of the products’ physicochemical parameters and their ethanol and residual solvents composition in view of the USP-NF (USP 43-NF 38) requirements. In addition, our research seeks to address ethylene oxide and gluten contamination of these products.

## 2. Results and Discussion

In our previous study [17], we presented LC/DAD/MS/MS method optimization and validation for the simultaneous determination of 13 biologically active ingredients in dietary supplements and raw materials used in the treatment of IBD. The content of curcuminoids varied from 15 to 102 mg/g samples. Green chiretta samples contained from 12 to 44 mg/g of andrographolides, while Indian frankincense samples contained less than 701 mg/g of boswellic acids. Regarding content analysis, our results highlighted the poor quality of several investigated products. These samples were subjected to further testing of their quality, including physicochemical characterization and the content of hazardous substances. 

### 2.1. Physicochemical Characterization 

The results of the evaluation of dietary supplements for the treatment of IBD (Table S1) physical parameters in view of the USP 43-NF 38 requirements (weight uniformity, tablet breaking force, friability, disintegration time, and rapture test) are presented in Table 1 (for the hard-shell capsule forms), Table 2 (for the soft-shell capsule forms), and Table 3 (for the tablet forms). 

To ensure the consistency and homogeneity of dosage units, uniformity in the weight study is an essential step in the quality control of dietary supplement products. Therefore, all investigated solid dosage forms (S1–S35) were characterized by broad net weight values in the range of 400.7–1191.9 mg for hard-shell capsules, 589.8–1581.8 mg for soft-shell capsules, and 224.5–1958.7 mg for tablets (Table 1, Table 2 and Table 3). The FDA guidance on solid dosage form design [18] reports that the size of tablets and capsules affects a patient’s compliance and acceptability of medication regimens due to swallowing difficulties and prolonged esophageal transit time. 

However, the average net weight of two dietary supplement samples in the form of hard-shell capsules (S6 and S16), two soft-shell capsules (S26 and S28), and three tablets (S31, S34, and S35) exceeded 1 000 mg, which may directly affect a patient’s ability to swallow these particular products. The net weight labeled by the manufacturer on the finished product declaration was distinguished only on 45.7% of oral solid-dosage-form samples. The average net weight was compared with declared values, and it was in a wide range from 71.1% (S7) to 124.2% (S1). Moreover, five samples in the form of hard-shell capsules (S1, S2, S7, S11, and S21) and one in the form of a tablet (S35) demonstrated conspicuous weight difference from the declared values (more than 10%), which indicates the doubtful reproducibility of their production process. Still, following the requirements of the USP weight variation study, all investigated products were within the specified range, meeting the requirements specific to individual dosage forms.

Hardness and friability tests were performed on tablets (S30–S35) to evaluate the structural integrity, mechanical strength, and susceptibility to breakage of dietary supplement products, which are crucial factors during product manufacturing, packaging, transportation, handling, and storage (Table 3). The breaking force was in the range of 27.7 N to 248.5 N. It is apparent that multicomponent products (S31, S34, and S35) with a high number of ingredients (both herbal extracts and excipients) are of high average net weight (over 1617.5 mg), and have high breaking force (above 138.2 N), which implies that the stated products probably needed high compression pressures during the tableting process. The good quality of the dietary supplement′s final product should be defined with repeatable breaking force values. Unfortunately, our results presented scattered breaking force values. The difference between the lowest and the highest reported values of breaking force was up to 67 N (S34), with relative standard deviations (RSD) up to 12.4% (S32). The friability test was focused on breaking, chipping, and capping of dietary supplement tablets during any form of locomotion, such as packing or transportation. It included assessing the tablet content loss and the changes in product appearance. The percentage weight loss of investigated tablets was lower than 0.38% within triplicate measurement. Additionally, there was no visible evidence of tablet cracking, chipping, or breaking; thus, all tablet samples passed the friability test.

Disintegration testing was performed to evaluate the ability of the investigated tablets (S30–S35) to break down into smaller particles via the breakdown of interparticulate bonds forged during their compaction. In the case of hard- and soft-shell capsules (S1–S29), the disintegration of gelatin and polysaccharide shells was a critical prerequisite for the herbal material release. The majority of tested products passed the disintegration requirement set by pharmacopeia for specified dosage forms (Table 1, Table 2 and Table 3). Moreover, for 62.9% of samples, it was observed that all investigated dosage units disintegrated completely within 15 min. Only one sample (S35—tablet form) did not disintegrate within 30 min. Moreover, none of its dosage units disintegrated, even after the continuation of the experiment for an additional 15 min (a total experimental time of 45 min). The high average weight (1958.7 mg) of this multicomponent product with a high breaking force value (138.2 N) can explain the observed negative results of the disintegration test.

Recent studies [19] have reported the prominent loss of quality of soft-shell capsules during storage in various conditions. Gelatin, the most widely used ingredient of soft-shell capsules, in certain storage conditions or the presence of specific compounds present as dietary supplement ingredients or final packaging components, may cause gelatin cross-linking. A pellicle, a thin water-insoluble transparent membrane of cross-linked protein, may form on the inner or outer surface of the capsule blocking the capsule fill from being released. To obtain meaningful data, all soft-shell capsule products were stored according to the manufacturer’s instructions and analyzed within the date of experience (S26–S29). All investigated soft-shell capsules met the requirements of the USP rupture test, as leakage of ingredients from shells was visible within a couple of minutes (Table 2).

The variations in the above-described parameters motivated us to evaluate the bulk and tapped density of the powdered mono herbal dietary supplement ingredients (S38–S49) (Table 4). There are various challenges currently being faced by dietary supplement industries due to the challenging flowability of multicomponent herbal powders. The flow property of the powders is the key to the success of tableting and capsule-filling. If the formulation is too free-flowing, unwanted segregation of the blend components can occur. On the other hand, if the material is too cohesive, then segregation is no longer a problem, but poor flow, blockage, unstable discharge, and subsequent stoppage of the equipment and processes may occur instead. The bulk and tapped density test was a convenient method that could be used to evaluate the herbal powder flow characteristics. The Hausner ratio and Compressibility Index were used as indirect methods of quantifying powder flowability from bulk and tapped density. These tests revealed that all investigated dietary supplements ingredients with a Hausner ratio higher than 1.32 and Compressibility Index higher than 24.3% (S43) might present flow challenges on the tablet-pressing and capsule-filling processes that can cause final product quality issues such as weight variability, content uniformity, and/or tablet defects. The most challenging herbal material for tableting-and capsule-filling processes was green chiretta, with a Hausner Ratio and Compressibility Index up to 1.79 and 48.4% (S40). This cohesive material demands a well-thought and comprehensive approach to formulation design that can improve powder flow during the manufacturing processes and thus reduce tablet/capsule shortfalls.

### 2.2. Assessment of Contaminants

#### 2.2.1. Volatile Matter Content

Purity tests, including loss upon drying, are important parameters to ensure that dietary supplements are free from unwanted materials. The test and requirements to ensure the absence of volatile matter present in hard-shell capsules (S1–S25), tablets (S30–S35), or bulky materials (S36–S51) are prescribed in each monograph under the Specific Tests monograph’s section as Limit Tests. Following the USP monograph, the assay limits are set as the loss of mass expressed as percent *w/w* with acceptance criterion based on the botanical source and sample type (extract or dried extract). Loss on drying was found to be up to 34.7% (S22) for tablet forms, 12.1% (S34) for hard-shell capsules, and 8.65% (S44) for dietary supplement ingredients (Table 5). The most intriguing weight loss of samples S22 and S34 may be related to the high amounts of hyaluronic acid in those samples. It is important to note that dietary supplement ingredients showed significant variation in moisture content in the triplicate sampling and analysis (RSD values were up to 47.64%). The bulky material was shown to be hygroscopic and moisture-reactive, but still, seven products were packaged in PVC foil with a poor barrier against moisture ingress. This finding confirms the usefulness of the quality of the packaging of raw materials in the quality of herbal dietary supplements. Finally, the weight loss of 10 samples (almost 20% of all samples), 4 in the form of the hard-shell capsule, 3 in the form of the tablet, and 3 powered dietary supplement ingredients, was not within USP specified limit.

**Figure 1:**
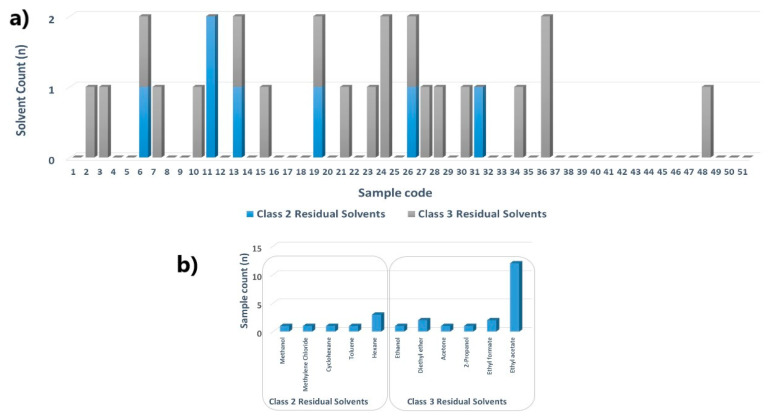
Evaluation of Class 2 and Class 3 residual solvents in dietary supplements in solid-dosage forms and dietary supplement ingredients: the number of residual solvents detected per sample (**a**); the number of samples containing specific residual solvent (**b**).

As the applied loss on drying test investigated the amount of volatile matter of any kind, we extended our research towards the determination of 47 residual solvents (Class 2 and Class 3) in solid dosage forms (S1–S35) and dietary supplement ingredients (S36–S51) (Table S2). First, the chromatographic system suitability was confirmed; the resolution between acetonitrile and methylene chloride in the Class 2 Mixture A Standard Solution was more than 1.0. Although the identified residual solvents were below pharmacopeia-specified levels (50–5000 ppm), it is intriguing to note that in almost 40% of analyzed samples, traces of Class 2 and Class 3 solvents were detected (Figure 1a). Moreover, more than half of oral dosage forms (51.4%) contained at least one of Class 2 and Class 3 residual solvents, while only 12.5% of dietary supplement ingredients as raw material were contaminated with Class 3 solvents. Ethyl acetate was the most detected solvent, present in almost 25% of all products (Figure 1b). There were up to two detectable residual solvents per product.

#### 2.2.2. Ethanol and Its Impurities Content

**Figure 2:**
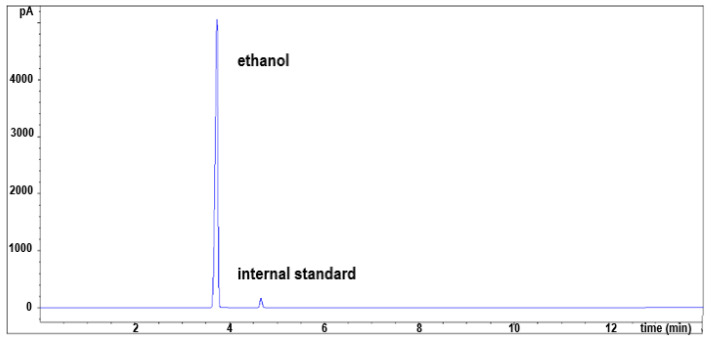
HSS-GC-FID chromatogram of sample S53.

Although many manufacturers of dietary supplements try to reduce the content of ethanol in liquid products or replace it with less hazardous and noxious extraction solvents, there are still many tinctures containing up to 70% ethanol on the market [14]. To ensure the safety of these products, the content of ethanol must be evaluated and should correspond to the declared value. The developed and validated HSS-GC-FID method was used to evaluate the content of ethanol and its main impurities in two liquid samples (S52 and S53). According to the information from the manufacturer, sample S52 contained 22% ethanol, while the manufacturer of product S53 gave broad information about alcohol content on the product label specifying content between 40% and 50%. The ethanol content found in the samples agreed with the labeled values, with impurities amounts far below toxic levels (Figure 2, Table 6). The daily amounts of ethanol that patients would ingest if they followed the daily servings recommended by the manufacturer are also presented in Table 6. 

Although the products contain high levels of ethanol, they are used at small doses (lower than 3 mL per daily serving). Therefore, the amounts of ethanol taken by daily servings were up to 1.41 mL. The total amount of ethanol present in each dietary supplement is also of concern since young children are at risk of unintentional ingestion of the total content because of careless placement of products by adults. The total amounts of ethanol in the samples were 8.1 mL (S52) and 23.5 mL (S53). The toxic dose in infants and young children is 0.4 mL/kg or 4.8 mL of ethanol for a 2-year-old child of 12 kg, while the life-threatening dose is expected to cause a deep coma with respiratory depression in a child of the same age, and weight is estimated at 48 mL. Our results cast a new light on the importance of dietary supplement packaging. According to the European Medicinal Agency recommendation, all products with greater than 5% ethanol content are required to have child-resistant closures [20]. Unfortunately, neither one of the products was closed with safety cap locks.

#### 2.2.3. Ethylene Oxide Content

Contamination of herbal material with pathogenic bacteria and fungi is a well-recognized problem attributed to growing conditions and the environment, as well as a lack of good agricultural and manufacturing practices within some producers. Ethylene oxide sterilization is one of the most efficient methods for the low-temperature sterilization of herbal material and packaging components for dietary supplement products. Unfortunately, it is by now generally accepted that ethylene oxide produces genetic damage in the somatic cells of exposed humans. This may be the reason why this technology has recently been subject to safety concerns. Recently, the contamination of herbal dietary supplements with ethylene oxide has been an outstanding problem; the excessive concentration of its residues was detected in multiple herbal product recalls. The most recent European Union Commission Regulation (EC) 2022/1396 states that no residue above 0.1 mg/kg of ethylene oxide is supposed to be present in products listed in EU legislation [21]. Because ethylene oxide represents a serious threat to the safety of IBD patients taking daily dietary supplements, we applied the advanced gas chromatography–tandem mass spectrometry (GC/MS/MS) technique to evaluate ethylene oxide contamination in solid (S1–S35) and liquid (S52 and S53) dietary supplement dosage forms as well as dietary supplement ingredients (S36–S51). Results are reported as EO_Total_ according to EC 2015/868 definition and expressed as ethylene oxide equivalents or the sum of native ethylene oxide and its by-product 2-chloroethanol obtained by reaction of acetaldehyde and chloride ion (Table 7, Figure S1). Extensive results carried out show that detectable levels of EO_Total_ were found in almost half of the samples. One concern about these findings was that eight samples contained levels above the EU permissible limit. It is important to highlight that all of them were purchased as dietary supplements dosage forms (seven in the form of hard-shell capsules and one in the form of a tablet). We speculate that this might be due to the sterilization of the primary packaging material in contact with the product, e.g., shells and blisters. This finding provides references for regulatory bodies to routinely control the quality of packaging material regarding ethylene oxide residues. Furthermore, the most alarming fact is that the levels found in two samples (S15 and S22) were up to 30 times higher than the EU limit. From this standpoint, these findings provide solid support for the stringent monitoring of ethylene oxide in dietary supplement finished products, packaging, and raw material.

#### 2.2.4. Gluten Content

Gluten refers to a complex, high-molecular-weight seed storage protein from grass-related grains, such as wheat, barley, and rye. Gluten-related disorders represent a spectrum of medical diseases classified based on pathogenesis as IgE-mediated wheat allergy, celiac disease, and non-celiac gluten sensitivity. It is estimated that gluten-related disorders affect up to 10% of the general population. Although some studies showed the similar prevalence of these disorders among IBD patients to that of the general population, there is increasing evidence that gluten can trigger an innate and adaptative immune response responsible for intestinal inflammation. Moreover, nearly one-third of IBD patients reported a diagnosis of non-celiac gluten sensitivity, and thus many follow a gluten-free diet. Notably, the recent data are consistent that celiac disease, due to genetic predisposition, is a risk factor for IBD, but an existing diagnosis of IBD is less likely associated with an increased risk for celiac disease [22,23,24]. While patients with gluten intolerance are usually aware of their condition and intake restrictions, there is a possibility of unintentional intake via dietary supplements since these may contain gluten in the formulation itself as an ingredient of some excipients or because of the cross-contamination during the manufacturing process in facilities using shared equipment. Thus, we applied an efficient, rapid, and reliable assay of gluten screening to detect traces of gluten in herbal dietary supplements used in the treatment of IBD (S1–S53). Samples were categorized into the following categories: gluten-free products with ˝Cross Grain˝ symbol (1 sample), products labeled as gluten-free (11 samples), gluten-free products by origin (2 samples), products reporting no information of gluten content (38 samples), and products reporting that they may contain traces of gluten (1 sample). Overall, 14 samples (26%) produced by different manufacturers were found positive for gluten (Table 8). In the group of the products labeled as gluten-free, three samples (S17, S20, and S39) were found to have gluten levels higher than the limit of quantitation but lower than the FDA restriction of 20 ppm. A total of 32% of the hard-shell capsules, 25% of soft-shell capsules, and 50% of tablet samples contained detectable levels of gluten. With regard to the product declaration of these products, no information on gluten content was given, and even more troublesome, two of them (S17 and S20) were labeled as gluten-free products.

## 3. Materials and Methods

### 3.1. Chemicals

USP Stock Standard Residual Solvents Class 2—Mix A, USP Stock Standard Residual Solvents Class 2—Mix B, and Residual Solvents Class 3 Mix A were obtained from United States Pharmacopeia (Rockville, MA, USA). Organic solvents acetone, acetonitrile, 1-butanol, ethanol, isobutanol, isopropanol, methanol, 1-propanol, and tert-butanol, all HPLC grade, were obtained from Merck (Darmstadt, Germany). Sodium acetate trihydrate for analysis (EMSURE^®^ ACS, ISO, Reag. Ph Eur), glacial acetic acid (100% Suprapur^®^), dimethylformamide (DMF) (for HPLC, purity ≥ 99.9%), and 2-chloroethanol D4 (98%) were purchased from Sigma-Aldrich (St. Louis, MO, USA). Analytical standards of ethylene oxide (50 mg/mL in methanol) and 2-chloroethanol (2 mg/mL in methanol) were available in ready-to-use solutions from Sigma-Aldrich (St. Louis, MO, USA). Gluten Detection Kit (Gluten-Tec competitive ELISA) was obtained from EuroProxima B.V. (Arnhem, the Netherlands). Ultra-pure water was obtained using a MilliQ UF-Plus system (resistivity MΩcm^−1^ > 18 at 25 °C and TOC < 5 ppb) from Millipore (Darmstadt, Germany). 

### 3.2. Samples

A total of 53 commercially available dietary supplement dosage forms (S1–S35, S52–S53) and dietary supplement ingredients (S36–S51) prepared from turmeric, Indian frankincense, green chiretta, and black pepper were obtained from various local pharmacies, health food stores, and online shops. The samples were coded and stored according to the manufacturers’ instructions. All samples were analyzed before the stated expiry date. A detailed description of all samples is given in Table S1, which is prepared based on the information available on the supplement packaging. The analyzed samples consisted of 25 dietary supplements formulated as a hard-shell capsule (S1–S25), 4 as a soft-shell capsule (S26–S29), 6 as a tablet (S30–S35), and 2 samples in the form of a tincture (S52–S53). A total of 16 samples were dietary supplement ingredients in the form of rhizome powder, leaf powder, resin extract, and oleoresin. These products were produced by 37 manufacturers from 15 different countries. The expiry date, when declared, varied from 2023 to 2024, and all of the samples were tested before their expiry date. The dietary supplements contained different plant products and plant-processed forms, i.e., dried extract, soft extract, liquid extract, resin, lyophilizate, and micellar solution. A total of 19 samples are available as a mono-herbal dietary supplement, while 16 included several plant species in their formulations.

### 3.3. Physicochemical Characterization

#### 3.3.1. Weight Variation of the Dosage Forms

The dietary supplements in the form of a hard-shell capsule, a soft-shell capsule, and a tablet were subjected to the weight variation test described in the USP 43-NF 38 guideline, <2091> chapter “Weight variation of dietary supplements” [25]. The test was performed using an analytical balance with a readability of 0.001 mg (MX5, Mettler Toledo, Greifensee, Switzerland). A total of 20 intact capsules of each hard-shell capsule sample (S1–S25) were individually weighed, and the average weight was calculated. Each capsule for which each of the individual weights was within the limits of 90% and 110% of the average weight met the pharmacopeia requirements. The protocol for soft-shell capsules (S26–S29) included weighting of intact capsules individually to obtain their gross weights. Afterward, each capsule was coded and cut open using a clean, sharp open blade, and the content was removed by washing with ultra-pure water. The occluded solvent was evaporated from the shells at the ambient temperature for 30 min. Each shell was weighted, and the net content was calculated. The pharmacopeia requirements are equivalent to those for hard-shell capsules. A total of 20 whole tablets of each uncoated tablet sample (S30–S35) were individually weighted, and the average weight was calculated. The USP requirements were fulfilled if the weights of not more than two of the tablets differed from the average weight by more than 5%, 7.5%, and 10% for tablets having an average weight lower than 130 mg, from 130 mg through 324 mg, and more than 324 mg, respectively, and no tablet differed in weight by more than double the stated percentage.

#### 3.3.2. Friability Test

The friability of tablets (S30–S35) was conducted following the USP 43-NF 38 guideline, <1216> chapter “Tablet friability” [26] using PTF 100 instrument by Pharma Test (Hainburg, Germany) and an analytical balance with a readability of 0.01 mg (AG254, Mettler Toledo, Greifensee, Switzerland). For tablets with a unit weight equal to or less than 650 mg, a sample of whole tablets corresponding as near as possible to 6.5 g was taken. For tablets with a unit weight of more than 650 mg, a sample of 10 whole tablets was taken. The tablets were carefully de-dusted before placing them in the drum. The drum was rotated 100 times. Any loose dust from tablets was removed, and tablets were visually inspected and accurately weighted. The USP requirements were fulfilled if the weight loss was not more than 1%. Samples were analyzed in triplicate.

#### 3.3.3. Tablet Breaking Force

The breaking force of tablet samples (S30–S35) was determined according to USP 43-NF 38 guidelines, <1217> chapter “Tablet Breaking Force” [27] on tablet hardness tester TBH 125 (Erweka, Germany). The breaking force that caused tablet fracture was carried out for ten tablets of each sample.

#### 3.3.4. Disintegration Time

The disintegration of hard- (S1–S25) and soft-shell (S26–S29) capsules and tablets (S30–S35) was determined using disintegration tester PTZ-s from Pharma Test (Hainburg, Germany), and the testing protocol described in USP 43-NF 38 guidelines, <2040> chapter “Disintegration and dissolution of dietary supplements” [28]. Six tablets/capsules of each dietary supplement were placed in each of the tubes of the basket and covered with disk. The specified medium (ultra-pure water for tablets and 0.05 M acetate buffer for capsules) at 37 ± 2 °C was used as the immersion fluid. After 30 min, the basket was lifted, and samples were observed to follow USP acceptance criteria for specified dosage forms that stated a disintegration time of less than 30 min.

#### 3.3.5. Rupture Test for Soft-Shell Capsule

For soft-shell capsules (S26–S29), USP <2040> chapter [28] also specifies a rupture test using a type II dissolution apparatus. USP 2 dissolution apparatus LDLT-A10 (Labtron Equipment Ltd., Fleet, UK) was used with the paddle rotation speed fixed at 50 rpm and bath thermostated at 37.0 °C with 500 mL ultra-pure water as a medium. According to USP acceptance criteria, all 6 tested dosage units should rupture within 15 min or not more than 2 of the total 18 capsules tested in more than 15 but not more than 30 min.

#### 3.3.6. Bulk and Tapped Density of Powders

The density of bulk samples was determined using tapped density testing instrument PT-DT300 from Pharma Test (Hainburg, Germany). First, 500 g of sample material was passed through a laboratory sieve with apertures of 1.0 mm (Retsch, Hann, Germany). The bulk and tapped densities were determined for each dietary supplement ingredient (S36–S51) according to the protocol described in USP 43-NF 38 guidelines, <616> chapter “Bulk Density and Tapped Density of Powders” in triplicate [29]. For each test, approximately 100 g of sample was carefully poured into a graduated 250 mL volumetric cylinder (readable to 2 mL) without compacting. The unsettled apparent powder volume was measured to the nearest unit, and the weight of the powder was determined. To obtain the tapped density, the mechanical tapped density testing instrument was set at a fixed drop of 14 ± 2 mm at a nominal rate of 300 drops, conforming to USP Method I. 10, 500, and 1250 taps were carried out, and the tapped volume was measured to the nearest density. When necessary, tapping was repeated an additional 1250 times, and the tapped volume was measured to the nearest unit. When the difference between the two values was lower than 2%, the test was done.

#### 3.3.7. Loss on Drying

The contents of dietary supplement dosage forms were combined before loss on drying evaluation to ensure homogeneity. A total of 20 dosage units of each hard-shell capsule (S1–S25) were opened to collect powder; the samples were ground if necessary. A total of 20 dosage units of each tablet (S30–S35) were powered using a porcelain mortar and pestle. A total of 10 g of powder and resin samples (S36–S51) was ground into a fine powder and mixed thoroughly. The samples were weighed (about 1.0 g) using the analytical balance AG254 (Mettler Toledo, Greifensee, Switzerland) and distributed on the aluminum sample plates (90 mm) as evenly as practicable to a depth of about 5 mm before being transferred into moisture analyzer DBS-60-3 (Kern and Sohn GmbH, Balingen, Germany) according to the protocol described in USP 43-NF 38 guidelines, <731> chapter “Loss on drying” in triplicate [30]. The procedure was performed at a temperature of 105 °C until constant weight. 

### 3.4. Assessment of Residual Solvents by HSS-GC-FID Method

#### 3.4.1. Standard Solutions and Sample Preparation

To evaluate the presence of residual solvents according to the protocol described in USP 43-NF 38 guidelines, <467> chapter “Residual Solvents” [31], three solutions containing all of Class 1 and Class 2 components listed in Table S2 were prepared. First, 1.0 mL of USP Stock Standard Residual Solvents Class 2—Mix A was diluted in a 100 mL glass volumetric flask with ultra-pure water. Class 2 Mixture A Standard Solution was prepared by adding 1 mL of prepared solution and 5 mL of ultra-pure water into a crimp top 20-mL headspace vials (Agilent Technologies, Santa Clara, CA, USA). Afterward, 1.0 mL of USP Stock Standard Residual Solvents Class 2—Mix B was diluted in a 100 mL glass volumetric flask with ultra-pure water. Class 2 Mixture B Standard Solution was prepared by adding 5 mL of prepared solution and 1 mL of ultra-pure water into crimp-top 20 mL headspace vials. Finally, 1.0 mL of USP Stock Standard Residual Solvents Class 3 Mix A was diluted in a 100 mL glass volumetric flask with ultra-pure water. Class 3 Mixture A Standard Solution was prepared by adding 1 mL of prepared solution and 1 mL of ultra-pure water into crimp-top 20 mL headspace vials. All final working solutions were immediately sealed with a polytetrafluoroethylene-lined septum and an aluminum crimp cap (Figure S2). 

A total of 20 dosage units of each hard-shell capsule sample (S1–S25) were opened to collect powder; the samples were ground if necessary. A total of 20 dosage units of each tablet sample (S30–S35) were powered using a porcelain mortar and pestle. A total of 100 g of powder and resin samples (S36–S51) was ground into a fine powder and mixed thoroughly. A total of 250 mg of powdered sample was transferred to a 25 mL volumetric flask and dispersed in DMF. A total of 5.0 mL of prepared sample was added to 1.0 mL of DMF in a 20 mL headspace vial, followed by sealing with polytetrafluoroethylene-lined septum and an aluminum crimp cap.

#### 3.4.2. Assay Protocol

Analysis was performed using Gas Chromatograph (GC) model 6850 equipped with Flame Ionization Detector (FID) and ChemStation data processing software, G1888 Headspace Sampler (HSS), all from Agilent Technologies, Santa Clara, CA, USA. The HSS oven temperature was set to 105 °C, with a 1 mL sample loop at 110 °C and HSS transfer line at 150 °C. The vial equilibration time was set to 40 min, with a pressure equilibration time of 30 s and an injection time of 1 min. All solvents were separated on a chromatographic column DB-624 (length—30 m, inner diameter—0.53 mm, and film thickness—5 μm) from Agilent Technologies, Santa Clara, CA, USA. The GC oven was maintained at 40 °C for 20 min, followed by a temperature ramp of 10 °C per minute to 240 °C, and maintained at 240 °C for 20 min. The carrier gas was nitrogen with a linear velocity of 5 mL/min and a split ratio of 1:5. The injection port and FID temperatures were kept at 140 °C and 250 °C, respectively [31].

### 3.5. Assessment of Ethanol and Its Impurities Content in Liquid Extracts using the HSS-GC-FID Method

#### 3.5.1. Standard Solutions and Sample Preparation

A stock solution of organic solvents (acetone, 1-butanol, ethanol, isobutanol, isopropanol, and 1-propanol, tert-butanol) and methanol was prepared using ultra-pure water in a concentration of 1.0% (*v/v*) and 0.6% (*v/v*), respectively. The stock solution of internal standard (acetonitrile) was prepared using ultra-pure water in a concentration of 0.1% (*v/v*). All stock solutions were kept at 4 °C while working solutions of ethanol and its impurities were freshly prepared in 20 mL HSS vials at the beginning of each day by the addition of an appropriate amount of the stock solution, internal standard, and dilution with ultra-pure water up to 2.0 mL.

Dietary supplements in liquid dosage forms (S52–S53) were shaken and unsealed before analysis. For ethanol evaluation, samples were diluted with ultra-pure water. A total of 2.0 mL of sample solution together with internal standard was transferred into 20 mL HSS vials. 

Before analysis, all HSS solutions were immediately sealed with a polytetrafluoroethylene-lined septum and an aluminum crimp cap.

#### 3.5.2. Assay Protocol

Analysis was performed using the same HSS-GC-FID instrumentation mentioned above. The HSS oven temperature was set to 90 °C, with a 1 mL sample loop at 110 °C and the HSS transfer line at 115 °C. The vial equilibration time was set to 15 min, with a pressure equilibration time of 1 min and an injection time of 1 min. All analytes were separated on a chromatographic column DB-624 (length—30 m, inner diameter—0.53 mm, and film thickness—5 μm) from Agilent Technologies (Santa Clara, CA, USA). The GC oven was maintained at 40 °C for 4 min, followed by a temperature ramp of 20 °C per minute to 60 °C, and maintained at 60 °C for additional 2 min, followed by a temperature ramp of 15 °C per minute to 180 °C. The carrier gas was nitrogen, with a linear velocity of 5 mL/min. The injection port and FID temperatures were kept at 230 °C and 300 °C, respectively. 

The method was validated according to the International Council for Harmonization of Technical Requirements for Pharmaceuticals for Human Use (ICH) guideline Q2 (R2) on validation of analytical procedures (Table S3, Figure S3) [32]. The examined parameters included selectivity, linearity (r ≥ 0.999), limits of detection (LOD ≤ 0.025%) and quantification (LOQ ≤ 0.075%), precision (RSD ≤ 5.27%), and accuracy (recovery from 93 to 110%).

### 3.6. Assessment of Ethylene Oxide using GC/MS/MS Method

#### 3.6.1. Standard Solutions and Sample Preparation

Due to the high volatility of ethylene oxide, the preparations of working solutions were performed at low temperatures. Therefore, all reagents and working solutions were kept cold before use. 

A total of 20 dosage units of each hard-shell capsule sample (S1–S25) were opened to collect powder; the samples were ground if necessary. A total of 20 dosage units of each tablet sample (S30–S35) were powered using a porcelain mortar and pestle. A total of 100 g of powder and resin samples (S36–S51) was ground into a fine powder. Dietary supplements in liquid dosage form (S52–S53) were shaken before analysis. Next, 2 g of each homogenized sample was added to a polypropylene tube, followed by the addition of 0.05 mL of internal standard (2-chloroethanol D4) and 0.95 mL of mixture acetonitrile:ultra-pure water (9:1, *v/v*). Samples were thoroughly mixed on a homogenizer (Mini GTM 1600, Spex Sample Prep, Metuchen, NJ, USA) and centrifugated for 10 min at 5000× *g* at −10 °C using a centrifuge (Z 32 HK, Hermle, Gosheim, Germany). In the final step, the samples were evaporated using a rotary evaporator (model EV311H from LabTech S.R.L., Sorisole, Italy). Finally, samples were reconstituted with 1 mL of acetonitrile and transferred into vials for GC–MS/MS analysis.

#### 3.6.2. Assay Protocol

Analyses were performed on gas chromatography coupled with tandem mass spectrometry (GC-MS/MS TQ8050, Shimadzu, Kyoto, Japan) and DB-17MS capillary column (length—20 m, diameter—0.180 mm, and 0.18 µm film thickness) from Agilent Technologies (Santa Clara, CA, USA). The GC oven gradient was set up as follows: 50 °C for 2 min amd a heat rate of 15 °C/min until 250 °C. The injection was performed in splitless mode, and the injection volume was 1 µL. The ionization of analytes was performed using electron impact ionization (EI) with a source temperature of 230 °C. The acquisition was performed using a tandem mass spectrometer in multiple reaction monitoring mode (MRM) by monitoring 2-chloroethanol: 80.0 → 31.0 (quantifier) and 82.0 → 31.0 (qualifier); internal standard 2-chloroethanol D4: 84.0 → 33.0 (quantifier) and 86.0 → 33.0 (qualifier). Data acquisition and subsequent data processing were carried out using LabSolution Software Version 4.52 (Shimadzu, Kyoto, Japan).

The method was validated according to the International Council for Harmonization of Technical Requirements for Pharmaceuticals for Human Use (ICH) guideline Q2 (R2) on validation of analytical procedures (Table S4) [32]. The examined parameters included selectivity, linearity (r = 0.996), limits of detection (LOD = 0.003 ppm) and quantification (LOQ = 0.009 ppm), precision (RSD ≤ 7.24%), and accuracy (recovery from 94 to 104%).

### 3.7. Assessment of Gluten using Enzyme-Linked Immunosorbent Assay

#### 3.7.1. Reagents and Sample Preparation

The competitive enzyme immunoassay for the quantitative determination of gliadin and gliadin fragments (Gluten-Tec competitive ELISA) was obtained from EuroProxima B.V. (Arnhem, the Netherlands). The kit and components were stored in a refrigerator at 4 °C until use. All kit components were brought to a dark place at ambient temperature before use. The rinsing and dilution buffers were diluted with ultra-pure water in a ratio of 1:20 and 1:4 (*v/v*), respectively. The concentrated conjugate (Peptide-HRP) solution was centrifugated for 1 min at 1000× *g* at ambient temperature using centrifuge model Z 326 K from Hermle (Gosheim, Germany), and in the next step, the supernatant was diluted 100 times using the dilution buffer. 

A total of 20 dosage units of each hard-shell capsule sample (S1–S25) were opened to collect powder; the samples were ground if necessary, while 20 dosage units of each soft-shell capsule sample (S26–S29) were cut open using a clean, sharp open blade; the content was removed from the shells, collected, and mixed thoroughly. A total of 20 dosage units of each tablet (S30–S35) were powered using a porcelain mortar and pestle. A total of 100 g of powder and resin samples (S36–S51) were ground into a fine powder and mixed thoroughly. The liquid samples (S52–S53) were shaken before sampling. A total of 0.5 g of homogenized sample was added to 4.5 mL of 60% ethanol solution and shaken for 20 min at 60 °C using water bath model WNB 22 with a shaking device and integrated PID-temperature controller (Memmert, Schwabach, Germany). The samples were centrifugated for 10 min at 2000× *g* at 20 °C using centrifuge model Z 326 K from Hermle (Gosheim, Germany). Afterward, the samples were diluted at a ratio of 1:10 with manufacturer’s wash buffer.

#### 3.7.2. Assay Protocol

The assay protocol was performed using an automatic microplate washer, PKL PPC 150 (PARAMEDICAL srl, Salerno, Italy). A total of 50 μL of the sample solution in duplicate was added to ELISA wells with 50 μL of diluted conjugate (Peptide-HRP) and left at 4 °C for 3 h to equilibrate with gentle agitation. ELISA wells were rinsed 3 times with wash buffer and tapped dry. A total of 100 μL of the substrate was added into each well and left to react at 25 °C for 30 min and stopped with 100 μL stop reagent (sulphuric acid). The absorbance at 450 nm was read on a plate reader (Das srl, Palombara Sabina, Italy) and calibrated using the standard solutions in duplicate. The absorbances from the plate reader were transformed into amounts of gliadin (Simplefit Excel file from EuroProxima B.V.) (Arnhem, the Netherlands). The linearity of the method was in the range of 0.15–5.0 ng/mL (r = 0.9994).

## 4. Conclusions

This work aimed to assess the physicochemical parameters of herbal dietary supplements used for the treatment of IBD. All products met the requirements of the USP weight variation test, friability, and rupture test. In contrast, one of the dietary supplements in the form of a tablet failed the USP disintegration test. The hardness test showed that multicomponent tablets containing several herbal extracts have high breaking force, implying that the stated products probably needed high compression pressures during the tableting process. Considering bulk and tapped density test results, it is evident that powered herbal raw materials are challenging during tableting and capsule-filling processes, with green chiretta extracts being the most cohesive. 

In addition, this study focuses on the assessment of contaminants such as moisture, residual solvents, ethylene oxide, and gluten. One concern about these findings was that eight samples contained levels of ethylene oxide above the EU permissible limit. Almost one-third of samples were found to be positive for gluten, but concentrations were lower than FDA restriction.

In conclusion, these findings add to a growing corpus of research showing the need for stricter dietary supplement quality control.

## Figures and Tables

**Table 1:** Physical parameters of the dietary supplements in hard-shell capsule form.

Sample Code	Weight Variation						Disintegration	
	Labeled Weight (mg)	Average Weight (mg, *n* = 20)	Percentage of Labeled Weight (%)	Minimum Percentage of Average Weight (%)	Maximum Percentage of Average Weight (%)	USP Criteria	Disintegration Time	USP Criteria
S1	400	496.8	124.2	95.6	104.4	passed	6 capsules within 15 min	passed
S2	500	601.1	120.2	93.6	105.4	passed	6 capsules within 15 min	passed
S3	600	600.3	100.1	94.7	106.9	passed	6 capsules within 15 min	passed
S4	633	649.8	102.6	93.3	106.5	passed	6 capsules within 30 min	passed
S5	587	597.8	101.8	95.5	104.4	passed	6 capsules within 15 min	passed
S6	N/D ^1^	1101.0	N/A ^2^	94.3	105.3	passed	6 capsules within 30 min	passed
S7	1167	830.2	71.1	98.6	101.6	passed	6 capsules within 15 min	passed
S8	500	499.6	99.9	97.7	104.2	passed	6 capsules within 15 min	passed
S9	N/D	579.4	N/A	92.6	107.1	passed	6 capsules within 30 min	passed
S10	N/D	507.8	N/A	89.5	106.5	passed	6 capsules within 30 min	passed
S11	500	400.7	80.1	91.1	105.5	passed	6 capsules within 30 min	passed
S12	N/D	527.7	N/A	97.6	108.4	passed	6 capsules within 30 min	passed
S13	N/D	563.8	N/A	100.0	100.0	passed	6 capsules within 30 min	passed
S14	N/D	408.9	N/A	98.0	102.8	passed	6 capsules within 30 min	passed
S15	N/D	486.5	N/A	98.3	101.7	passed	6 capsules within 15 min	passed
S16	N/D	1191.9	N/A	94.6	102.9	passed	6 capsules within 15 min	passed
S17	N/D	484.1	N/A	95.3	108.5	passed	6 capsules within 30 min	passed
S18	N/D	594.5	N/A	96.0	104.9	passed	6 capsules within 15 min	passed
S19	N/D	532.1	N/A	94.2	105.9	passed	6 capsules within 15 min	passed
S20	N/D	542.7	N/A	94.4	107.7	passed	6 capsules within 30 min	passed
S21	500	597.7	119.5	95.6	105.2	passed	6 capsules within 15 min	passed
S22	980	985.8	100.6	96.7	105.2	passed	6 capsules within 15 min	passed
S23	N/D	606.0	N/A	94.8	105.9	passed	6 capsules within 15 min	passed
S24	N/D	995.0	N/A	97.4	101.8	passed	6 capsules within 15 min	passed
S25	N/D	814.6	N/A	97.5	101.6	passed	6 capsules within 15 min	passed

^1^ N/D—no information on the package; ^2^ N/A—not applicable.

**Table 2:** Physical parameters of the dietary supplements in soft-shell capsule form.

Sample Code	Weight Variation						Disintegration		Rupture Test	
	Labeled Weight (mg)	Average Weight (mg, *n* = 20)	Percentage of Labeled Weight (%)	Minimum Percentage of Average Weight (%)	Maximum Percentage of Average Weight (%)	USP Criteria	Disintegration Time	USP Criteria	Rupture Time	USP Criteria
S26	N/D ^1^	1581.8	N/A ^2^	99.0	101.3	passed	6 capsules within 15 min	passed	6 capsules within 1 min	passed
S27	916.7	959.6	104.7	95.4	102.7	passed	6 capsules within 15 min	passed	6 capsules within 1 min	passed
S28	1280	1303.0	101.8	97.8	101.6	passed	6 capsules within 15 min	passed	6 capsules within 3 min	passed
S29	596	589.8	99.0	99.0	101.0	passed	6 capsules within 15 min	passed	6 capsules within 7 min	passed

^1^ N/D—no information on the package; ^2^ N/A—not applicable.

**Table 3:** Physical parameters of the dietary supplements in tablet form.

Sample code	Weight Variation						Disintegration		Tablet Breaking Force		Friability	
	Labeled Weight (mg)	Average Weight (mg, *n* = 20)	Percentage of Labeled Weight (%)	Minimum Percentage of Average Weight (%)	Maximum Percentage of Average Weight (%)	USP Criteria	Disintegration Time	USP Criteria	Hardness (N, *n* = 10)	RSD (%) ^1^	Friability (%, *n* = 3)	USP Criteria
S30	N/D ^2^	224.5	N/A ^3^	94.1	103.1	passed	6 tablets disintegrate completely within 15 min	passed	46.0	0.1	≤0.01	passed
S31	1633.3	1621.2	99.3	98.4	102.9	passed	6 tablets disintegrate completely within 30 min	passed	248.5	7.7	≤0.01	passed
S32	N/D	335.7	N/A	97.7	102.1	passed	6 tablets disintegrate completely within 1 min	passed	73.7	12.4	≤0.01	passed
S33	300	294.0	98.0	95.0	107.7	passed (1 unit > 7.5%)	6 tablets disintegrate completely within 5 min	passed	27.7	6.5	0.14–0.38	passed
S34	1680	1617.5	96.3	97.9	103.8	passed	6 tablets disintegrate completely within 25 min	passed	241.0	10.2	≤0.01	passed
S35	1584.5	1958.7	123.6	98.8	101.0	passed	none of the tablets disintegrate completely within 30 min	failed	138.2	7.2	0.1–0.28	passed

^1^ RSD—Relative Standard Deviation; ^2^ N/D—no information on the package; ^3^ N/A—not applicable.

**Table 4:** Bulk density, tapped density, Compressibility Index, and Hausner ratio of the dietary supplement ingredients.

Botanical Source	Turmeric	Indian Frankincense	Green Chiretta	Black Pepper
bulk density (g/mL)				
Average	0.4778	0.4201	0.3172	0.4555
Median	0.4698	0.4196	0.3236	N/A ^1^
Range	0.4070–0.5525	0.2958–0.5329	0.3040–0.3240	N/A
tapped density (g/mL)				
Average	0.6569	0.6159	0.5847	0.6263
Median	0.6664	0.61946	0.5892	N/A
Range	0.5644–0.7375	0.4986–0.7190	0.5779–0.5892	N/A
Hausner ratio				
Average	1.38	1.50	1.84	1.38
Median	1.39	1.45	1.81	N/A
Range	1.32–1.45	1.35–1.69	1.79–1.92	N/A
Compressibility Index (%)				
Average	27.5	32.7	45.7	27.3
Median	28.0	33.0	44.8	N/A
Range	24.3–31.0	25.9–40.7	44.0–48.4	N/A
USP category	passable—poor	poor—very poor	very, very poor	poor

^1^ N/A—not applicable.

**Table 5:** Loss on drying of the dietary supplements in solid-dosage forms and dietary supplement ingredients.

Sample Type	Loss on Drying (%, *n* = 3)	RSD ^1^ (%)	USP Criteria
hard-shell capsule			
dry extract	1.05–34.67	≤11.01	17 passed, 4 failed
extract	6.32–8.04	0.21–5.54	4 passed
tablet			
dry extract	2.55–12.14	0.29–9.33	2 passed, 3 failed
extract	6.20	7.53	passed
dietary supplement ingredient (powder)			
dry extract	1.05–5.15	12.10–10.94	2 passed, 3 failed
extract	2.40–8.65	0.92–47.64	12 passed

^1^ RSD—Relative Standard Deviation.

**Table 6:** Content of ethanol (%) and its major impurities (ppm) in dietary supplements in the liquid dosage form.

Sample Code	Labeled Ethanol Content (%)	Ethanol (*n* = 3, %,/ RSD ^1^, %)	Amount of Ethanol Per Daily Serving (mL)	Methanol	Acetone	Isopropanol	Tert-Butanol	1-Propanol	Isobutanol	1-Butanol
				(n = 3, ppm/RSD, %)						
S52	22	16.21/4.56	0.19	10.64/1.99	<LOQ	8.59/2.53	<LOQ	<LOQ	<LOD	<LOD
S53	40–50	47.08/2.41	1.41	18.12/1.07	3.59/2.00	15.66/1.44	<LOQ	<LOQ	<LOD	<LOD

^1^ Relative Standard Deviation.

**Table 7:** Ethylene oxide content expressed as EO_Total_ in dietary supplement dosage forms and dietary supplement ingredients.

Sample Type	Incidence [%] ^1^ (Number of QUANTIFIED Samples)	Mean (mg/kg)	Median (mg/kg)	Range of Quantified Values (mg/kg)
hard-shell capsule	52.00 (13)	0.61	0.14	0.02–3.29
soft-shell capsule	25.00 (1)	0.05		
tablet	66.66 (4)	0.42	0.04	0.02–1.58
dietary supplement ingredient	43.75 (7)	0.37	0.02	0.02–0.09
tincture	0 (0)	N/A ^2^	N/A	N/A

^1^ Incidence—[(the number of quantified samples)/(number of total samples)] × 100; ^2^ N/A—not applicable.

**Table 8:** Gluten content in dietary supplement dosage forms and dietary supplement ingredients.

Sample Type	Incidence (%) ^1^ (Number of Quantified Samples)	Mean (ppm)	Median (ppm)	Range of Quantified Values (ppm)
Classification by dosage form				
hard-shell capsule	32.00 (8)	1.36	1.20	0.69–2.31
soft-shell capsule	25.00 (1)	N/A ^2^	N/A	5.97
tablet	50.00 (3)	1.11	1.06	0.57–1.70
dietary supplement ingredient	13.33 (2)	1.62	1.62	0.69–2.54
tincture	0 (0)	N/A	N/A	N/A
Classification by product label				
gluten-free products with “Cross Grain” symbol	0 (0)	N/A	N/A	N/A
products labeled as gluten-free	27.27 (3)	1.43	1.79	0.69–2.31
naturally (by origin) gluten-free products	0 (0)	N/A	N/A	N/A
products with unknown gluten content	28.95 (11)	1.74	1.11	0.57–5.97
products labeled that they may contain traces of gluten	0 (0)	N/A	N/A	N/A

^1^ Incidence—[(the number of quantified samples)/(number of total samples)] × 100; ^2^ N/A—not applicable.

## Data Availability

Data are contained within the article and Supplementary Materials.

## Supplementary Materials

The following supporting information can be downloaded at: https://www.mdpi.com/article/10.3390/ph16060893/s1, Figure S1: GC/MS/MS chromatogram of sample S35. Figure S2: Chromatogram of Class 2 Mixture A Standard Solution (a), Class 2 Mixture B Standard Solution (b), and Class 3 Mixture A Standard Solution (c). Figure S3: Chromatogram of standard solution (0.1 % (V/V)). Table S1: The manufacturers’ information given on the product label. Table S2: Residual solvents investigated in dietary supplements’ solid-dosage forms and dietary supplements’ ingredients according to the protocol described in USP 43-NF 38 guidelines, <467> chapter “Residual Solvents”. Table S3: HSS-GC-FID method validation data. Table S4: GC/MS/MS method validation data.
