# Supplementary material for: Assessment of Physicochemical Parameters and Contaminants in Herbal Dietary Supplements Used in the Treatment of Inflammatory Bowel Disease
Source: Pharmaceuticals (Basel). 2023 Jun 18;16(6):893. doi: 10.3390/ph16060893 (PMC10300715; doi:10.3390/ph16060893)
Supplement: Supplementary file 1 [file pharmaceuticals-16-00893-s001.zip › pharmaceuticals-2384671-supplementary.pdf]

Table S1. The manufacturers' information given on the product label.

Sample code	Sample type	Botanical source	Recommended daily dosage	Number of dosage units	Product net weight	Gluten content labelling	Origin county
S1	hard-shell capsule	Turmeric, Black pepper	2 capsules	60	24 g	not labelled	Croatia
S2	hard-shell capsule	Turmeric	2 capsules	60	30 g	not labelled	Croatia
S3	hard-shell capsule	Turmeric	2 capsules	60	not labelled	not labelled	Italy
S4	hard-shell capsule	Turmeric, Black pepper	1 capsule with meal	30	19 g	not labelled	Austria
S5	hard-shell capsule	Turmeric, Black pepper	2 capsules with meal	60	35.22 g	not labelled	Croatia
S6	hard-shell capsule	Turmeric, Black pepper	6 capsules 45 min before meal	60	not labelled	gluten-free	no information
S7	hard-shell capsule	Turmeric	1 capsule	66	77 g	gluten-free	Canada
S8	hard-shell capsule	Turmeric, Black pepper	2 capsules with meal	60	30 g	not labelled	Netherlands
S9	hard-shell capsule	Turmeric, Black pepper	6 capsules	60	not labelled	not labelled	Thailand
S10	hard-shell capsule	Turmeric	3 capsules after meal	100	not labelled	not labelled	Thailand
S11	hard-shell capsule	Indian frankincense	2 capsules	60	not labelled	not labelled	India
S12	hard-shell capsule	Indian frankincense	2 capsules with meal	60	not labelled	gluten-free by origin	India
S13	hard-shell capsule	Indian frankincense	2 capsules	60	not labelled	not labelled	UK
S14	hard-shell capsule	Indian frankincense	2 capsules	60	not labelled	gluten-free	India
S15	hard-shell capsule	Indian frankincense	1 capsule with meal	60	not labelled	not labelled	Croatia
S16	hard-shell capsule	Indian frankincense	2 capsules with meal	80	not labelled	not labelled	Croatia
S17	hard-shell capsule	Green chiretta	1 capsule	60	not labelled	gluten-free	India
S18	hard-shell capsule	Green chiretta	2 capsules	30	not labelled	not labelled	India
S19	hard-shell capsule	Green chiretta	4 capsules with meal	60	not labelled	not labelled	Thailand
S20	hard-shell capsule	Turmeric, Green chiretta	1 capsule with meal	60	not labelled	gluten-free	India
S21	hard-shell capsule	Turmeric, Black pepper	2 capsules after meal	60	30 g	gluten-free by origin	Croatia
S22	hard-shell capsule	Turmeric, Indian frankincense	2 capsules with meal	60	58.8 g	gluten-free	Croatia
S23	hard-shell capsule	Turmeric, Green chiretta	2 capsules	60	not labelled	not labelled	USA
S24	hard-shell capsule	Turmeric, Green chiretta	6 capsules with meal	60	not labelled	not labelled	USA
S25	hard-shell capsule	Turmeric, Indian frankincense	3 capsules	60	not labelled	not labelled	USA
S26	soft-shell capsule	Turmeric, Black pepper	1 capsule with meal	30	not labelled	not labelled	Croatia

S27	soft-shell capsule	Turmeric	8 capsules	60	55 g	not labelled	Belgium
S28	soft-shell capsule	Turmeric	1 capsule with meal	30	38.4 g	not labelled	USA
S29	soft-shell capsule	Turmeric	1 capsule with meal	30	17.8 g	not labelled	Croatia
S30	tablet	Turmeric, Black pepper	2 tablets before meal			not labelled	Thailand
S31	tablet	Turmeric	1 tablet with meal	30	49 g	not labelled	USA
S32	tablet	Turmeric	2 tablets			not labelled	UK
S33	tablet	Turmeric, Black pepper	10 tablets	300	90 g	gluten-free with ˝Cross Grain˝ symbol	Germany
S34	tablet	Indian frankincense	2 tablets with meal	40	67.2 g	not labelled	USA
S35	tablet	Turmeric, Indian frankincense	2 tablets before meal	60	Not labelled	not labelled	USA
S36	powder	Turmeric, Indian frankincense, Black pepper	not applicable	not applicable	100 g	not labelled	India
S37	powder	Turmeric, Black pepper	not applicable	not applicable	27 g	not labelled	Netherlands
S38	powder	Indian frankincense	not applicable	not applicable	100 g	gluten-free	India
S39	powder	Green chiretta	not applicable	not applicable	100 g	gluten-free	India
S40	powder	Green chiretta	not applicable	not applicable	100 g	gluten-free	India
S41	powder	Green chiretta	not applicable	not applicable	100 g	gluten-free	India
S42	powder	Black pepper	not applicable	not applicable	100 g	gluten-free	India
S43	powder	Turmeric	not applicable	not applicable	200 g	not labelled	Croatia
S44	powder	Turmeric	not applicable	not applicable	100 g	not labelled	Germany
S45	powder	Turmeric	not applicable	not applicable	100 g	not labelled	Croatia
S46	powder	Turmeric	not applicable	not applicable	100 g	not labelled	Croatia
S47	powder	Turmeric	not applicable	not applicable	100 g	gluten might be present in traces	Austria
S48	powder	Turmeric	not applicable	not applicable	50 g	not labelled	Croatia
S49	resin	Indian frankincense	not applicable	not applicable	100 g	not labelled	Greece
S50	resin	Indian frankincense	not applicable	not applicable	200 g	not labelled	Turky
S51	resin	Indian frankincense	not applicable	not applicable	100 g	not labelled	no information
S52	tincture	Turmeric	1.2 ml	not applicable	50 mL	not labelled	Bulgaria
S53	tincture	Turmeric	60 drops	not applicable	50 mL	not labelled	Croatia

Table S2. Residual solvents investigated in dietary supplements solid dosage forms and dietary supplements ingredients according to protocol described in USP 43-NF 38 guideline, <467> chapter "Residual Solvents".

**Residual Solvent**	**Retention time ($t_{R,}$ min)**	**Peak area (AUC, pA/s)**
**Class 2**		
methanol	2.90	19.58
acetonitrile	5.06	6.71
methylene chloride	5.36	73.26
trans-1,2-dichloroethene	5.95	398.13
cis-1,2-dichloroethene	8.67	265.43
tetrahydrofuran	9.72	53.82
cyclohexane	10.63	7161.08
methylcyclohexane	16.92	2275.34
1,4-dioxane	18.81	3.38
toluene	23.99	1003.53
chlorobenzene	28.22	202.04
ethylbenzene	28.55	446.10
m-xylene	28.84	1837.40
p-xylene	28.84	1837.40
o-xylene	29.74	179.80
hexane	6.59	1466.82
nitromethane	8.51	1.85
chloroform	9.84	19.10
1,2-dimethoxyethane	12.47	4.94
trichloroethane	15.76	94.95
pyridine	23.85	11.88
2-hexanone	26.49	20.71
tetralin	36.48	192.80
n-pentane	3.26	42.40
**Class 3**		

ethanol	3.27	11313.40
diethyl ether	3.83	93.00
acetone	3.99	3425.50
2-propanol	4.47	288.80
ethyl formate	4.74	720.00
methyl acetate	5.09	383.90
methyl *tert*-butyl ether	5.87	3655.00
1-propanol	7.31	158.10
2-butanone	8.66	386.30
ethyl acetate	8.94	571.70
2-butanol	9.53	246.40
isopropyl acetate	12.22	266.80
isobutanol	12.82	1204.00
n-heptane	13.60	14751.20
1-butanol	16.41	198.80
propyl acetate	19.68	1013.60
4-methyl-2-pentanone	23.50	1109.00
3-methyl-1-butanol	24.28	258.80
isobutyl acetate	24.93	1711.40
1-pentanol	25.95	226.30
butyl acetate	26.87	1316.90
dimethyl sulfoxide	27.78	11469.40
anisole	30.67	1031.60

Table S3. HSS-GC-FID method validation data.

**Analyte**	**Retention time (t_R, min/ RSD,[1] %)**	**Linearity range (ppm)** [2]	**Regression equation**	***r***[3]	**LOD**[4]	**LOQ**[5]
ethanol	3.73 / 0.24	1000-50000	y = 8.0136x + 0.3595	0.9998	0.025%	0.075%
methanol	2.77 / 0.37	3-3600	y = 0.00042x – 0.00050	0.9999	0.50 ppm	1.5 ppm
acetone	4.31 / 0.11	5-6000	y = 0.00193x – 0.00139	0.9999	0.50 ppm	1.5 ppm
isopropanol	4.53 / 0.08	5-6000	y = 0.00106x – 0.00657	0.9999	0.50 ppm	1.5 ppm
tert-butanol	5.06 / 0.37	5-6000	y = 0.00230x – 0.00500	0.9999	0.33 ppm	1.0 ppm
1-propranol	6.01 / 0.21	5-6000	y = 0.00092x – 0.00358	0.9999	0.50 ppm	1.5 ppm
isobutanol	7.62 / 0.24	5-6000	y = 0.00135x + 0.01049	0.9999	0.33 ppm	1.0 ppm
1-butanol	8.36 / 0.41	5-6000	y = 0.00087x + 0.00090	0.9999	0.33 ppm	1.0 ppm

**Analyte**	**Precision (RSD, %)**[6]		**Accuracy (Recovery and RSD, %)**[7]					
	**Repeatability (*n* = 6)**	**Intermediate precision (*n* = 9)**	**Low (*n* = 3)**		**Medium (*n* = 3)**		**High (*n* = 3)**	
ethanol	1.91	3.68	97.35	6.07	99.81	3.55	102.51	0.80
methanol	3.65	4.51	104.55	6.70	96.28	2.03	101.84	2.14
acetone	2.05	2.88	92.65	1.11	96.88	2.14	99.35	0.65
isopropanol	4.88	4.08	110.00	0.30	96.69	2.15	97.52	2.44
tert-butanol	1.67	4.81	109.65	3.82	104.39	4.76	101.08	2.94
1-propranol	2.95	3.48	104.72	2.85	96.26	1.49	96.78	3.49
isobutanol	2.38	2.87	101.11	3.05	101.57	1.75	96.80	3.40
1-butanol	4.76	5.27	93.65	4.40	97.36	2.87	95.71	4.28

[1]RSD – Relative Standard Deviation

[2]Linearity was examined on at least five concentration levels in three individual standard solution preparations from which a single regression line was constructed. For ethanol impurities the highest concentration level was 120% of the specification limit.

[3]*r* – correlation coefficient

[4]LOD – Limit of Detection was determined using signal-to-noise value 3.

[5]LOQ – Limit of Quantitation was determined using signal-to-noise value 10.

[6]Repeatability was assessed analysing six individual samples of working solution (repeatability level: 2.5% for ethanol, 300 ppm for methanol and 500 ppm for other impurities) on the same day, while intermediate precision was examined on three individual samples over three days. The data are expressed as RSDs.

[7]Accuracy of the method was examined by analysis of standard solutions in triplicate on three concentration levels (low: 0.1% ethanol, 15 ppm methanol, 25 ppm other impurities; medium: 2.5% ethanol, 300 ppm methanol, 500 ppm other impurities; high: 5% ethanol, 3600 ppm methanol, 6000 ppm other impurities). The data are expressed both as recoveries and RSDs.

Table S4. GC/MS/MS method validation data.

Analyte	Retention time (t_R, min/ RSD[1], %)	Linearity range (ppm) [2]	Regression equation	r[3]	LOD (ppm)[4]	LOQ (ppm)[5]
2-CE	2.80	0.02-0.2	y = 16470x + 44.757	0.996	0.003	0.009

Analyte	Precision (RSD, %)[6]		Accuracy (Recovery and RSD, %)[7]		
	Repeatability (n = 5)	Intermediate precision (n = 10)	Low (n = 3)	Medium (n = 3)	High (n = 3)
2-CE	2.60	7.24	97.11	94.13	103.88

[1]RSD – Relative Standard Deviation

[2]Linearity was examined on at least five concentration levels in three individual standard solution preparations from which a single regression line was constructed.

[3]r – correlation coefficient

[4]LOD – Limit of Detection was determined using signal-to-noise value 3.

[5]LOQ – Limit of Quantitation was determined using signal-to-noise value 10.

[6]Repeatability was assessed analysing five individual samples of working solution (repeatability level: 0.1 ppm) on the same day, while intermediate precision was examined on five individual samples over two days. The data are expressed as RSDs.

[7]Accuracy of the method was examined by analysis of standard solutions in triplicate on three concentration levels (low: 0.02 ppm; medium: 0.1 ppm; high: 0.2 ppm). The data are expressed both as recoveries and RSDs.

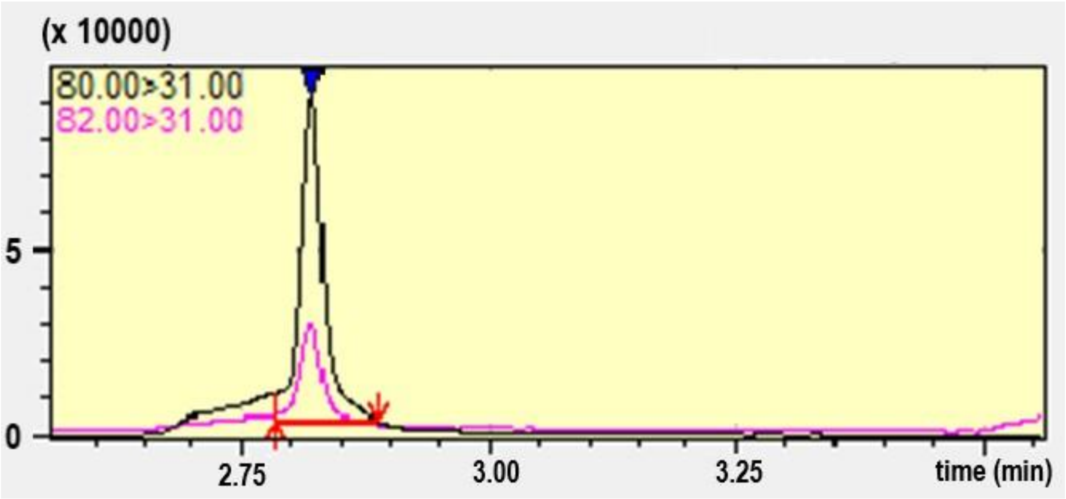

Figure S1. GC/MS/MS chromatogram of sample S35.

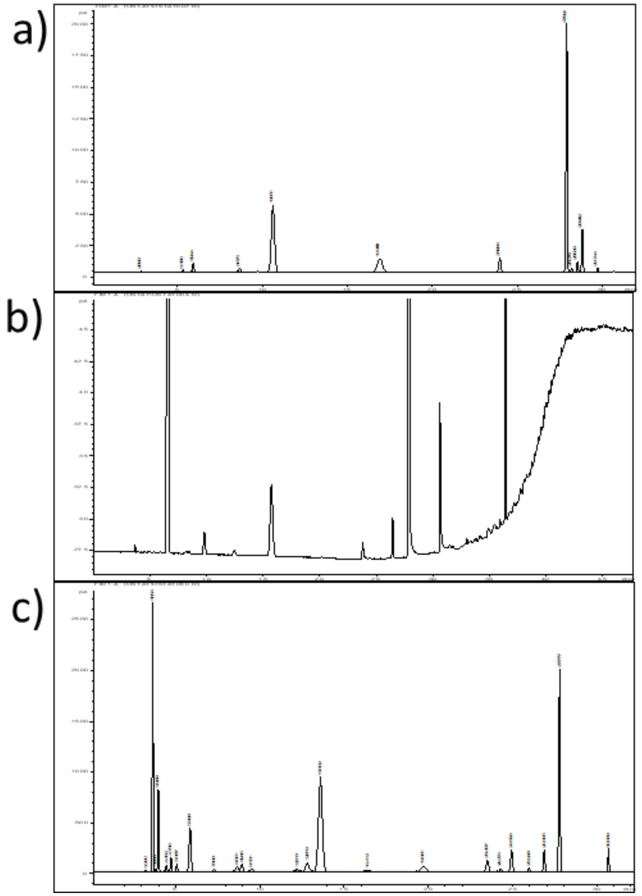

Figure S2. Chromatogram of Class 2 Mixture A Standard Solution (**a**), Class 2 Mixture B Standard Solution (**b**), and Class 3 Mixture A Standard Solution (**c**).

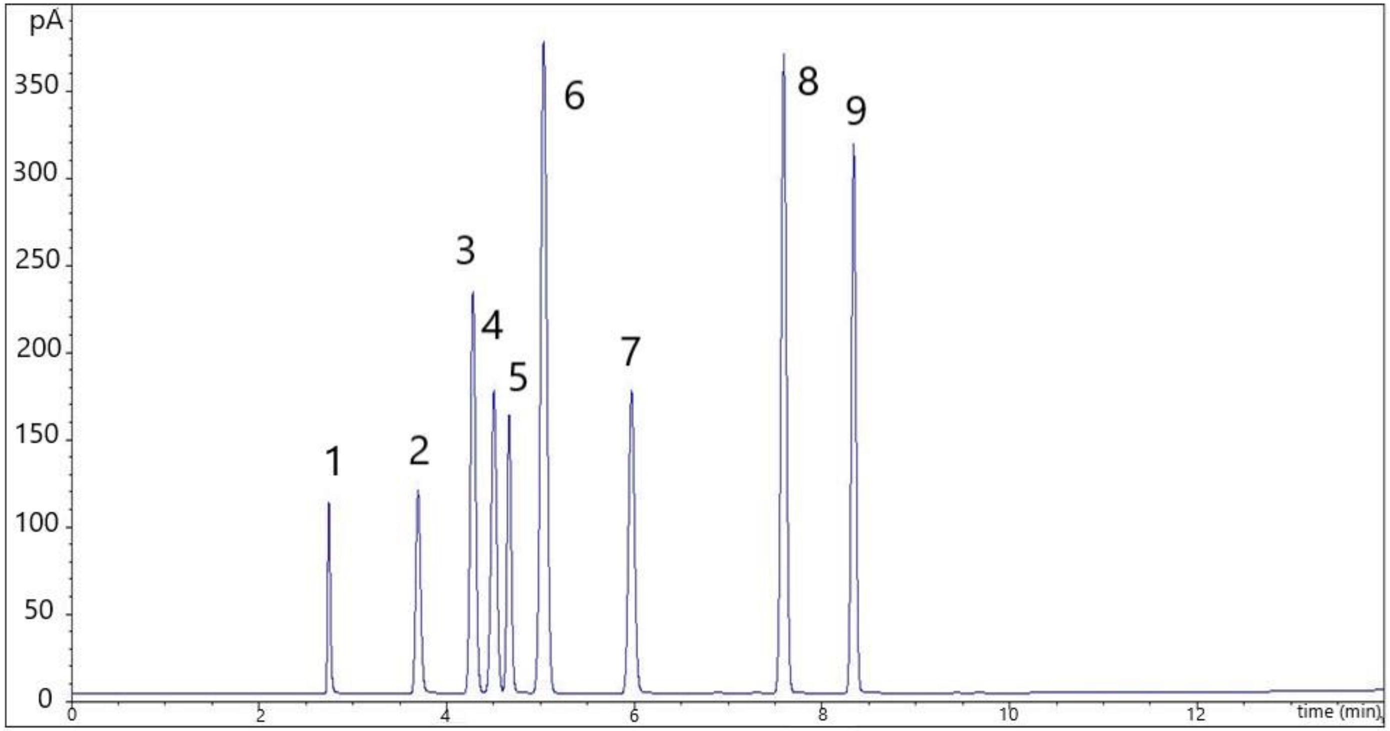

Figure S3. Chromatogram of standard solution (0.1 % (*V/V*)). Legend: methanol (1), ethanol (2), acetone (3), isopropanol (4), acetonitril – internal standard (5), *tert*-butanol (6), 1-propranol (7), isobutanol (8) i 1-butanol (9).
